# The Outcome of Old-School Indirect Pulp Capping over 40 Years: A Practice-Based Retrospective Evaluation

**DOI:** 10.3390/dj13050182

**Published:** 2025-04-22

**Authors:** Roland Frankenberger, Andreas Koch, Lina Plohmann, Benedicta Beck-Broichsitter, Stephan Becker

**Affiliations:** 1 Department of Operative Dentistry, Endodontics, and Pediatric Dentistry, Medical Center for Dentistry, University Medical Center Giessen and Marburg, Georg-Voigt-Str. 3, D-35039 Marburg, Germany; kochan@staff.uni-marburg.de; 2 Zahnarztpraxis Sommer, Hauptstraße 67, 23898 Sandesneben, Germany; lina.plohmann@gmail.com; 3 Clinic for Oral and Maxillofacial Surgery, Stuttgart City Clinic, Kriegsbergerstr. 60, 70174 Stuttgart, Germany; benedicta.beck.broichsitter@googlemail.com; 4 Becker & Kollegen, Kehdenstrasse 2-10, D-24013 Kiel, Germany; praxis@mkg-becker.de

**Keywords:** Indirect pulp capping, zinc eugenol, amalgam, resin composite, pulp vitality

## Abstract

(1) **Background:** The aim of this in vitro study was to evaluate the success of indirect pulp capping (IPC) procedures over a period of 40 years. (2) **Methods:** The investigation of 1412 dental records included 159 patients with 366 IPC teeth having been treated from 1969 to 1980. The teeth revealed caries within the inner third of dentin, were symptom-free, and showed no pulp exposure. The caries were excavated thoroughly and teeth with accidentally exposed pulp were excluded. Zinc–oxide–eugenol was used for the IPC procedures. The posterior teeth were restored with amalgam, and the anterior teeth received direct resin composite fillings. The gathered parameters with possible influences on survival rates were age, gender, tooth locations/positions, dates of vital therapy, the number of filled surfaces, types of primary restoration material, successional treatments on each tooth, and the last dates of surveillance. Data collection and statistical analysis were performed using Excel sheets and DataTab. Significant differences among groups were computed by cox regression analysis and the significance level was set at *p* = 0.05. Kaplan–Meier curves were utilized to illustrate the survival rates. (3) **Results:** Treatment success was measured by the maintenance of vitality beyond 365 days. The loss of vitality within 365 days was determined as treatment failure. Treatment outcomes were assessed after different time periods (1 and 6 months; 1, 2, 5, 10, 20, and 40 years). Pulp vitality dropped from 95% after 3 months to 32% after 40 years. Cavity size had a significant influence on the survival of pulp, but tooth position did not; however, third molars at least initially showed a better outcome. Beyond the 1-year recall, no differences for the evaluated parameters were present. (4) **Conclusions:** IPC showed excellent long-term success rates, revealing a 1.7% annual failure rate after 40 years of clinical service. Larger defects suffer more pulp damage in the long run.

## 1. Introduction

Today, tooth-colored dental restorations are predominantly bonded to the hard tissues of teeth [1]. Whereas successful enamel bonding guarantees overall retention, dentin adhesion provides an additional stabilization of the remaining tooth’s hard tissues and mainly helps to effectively seal the pulpo–dentin complex in order to successfully avoid postoperative hypersensitivities [1,2,3,4]. With this course of treatment, conventional cement liners, such as phosphate or glass ionomer cements, are not normally applied anymore, because they significantly reduce the dentin bonding area [1,2,5]. Furthermore, non-adhesive cements are time-consuming without providing additional value in terms of pulp vitality [1,5,6]. In contrast, decades ago conventional liners played a greater role—i.e., they were routinely applied beneath every cast gold or amalgam, and were even used in resin composite restoration [7]. Moreover, in cavities estimated to be very deep, special measures have been taken to preserve tooth vitality [8,9,10].

Today, IPC has been largely sidelined due to a fundamentally different understanding of caries excavation [11,12,13]. It has been scientifically proven that selective excavation is preferable to the old-fashioned, “complete”, and usually aggressive method of caries excavation [11,14]. As with most paradigm shifts, the introduction of this approach to caries excavation into dental practice is usually a slow and gradual process, which takes at least one generation of dentists to implement [13]. In the specific case of the excavation strategy, this is probably also due to the fact that many colleagues are afraid of being discredited as not working cleanly in the event that a patient changes dentist and the next operator detects misinterpreted “secondary caries”. Therefore, seemingly complete excavation is still widespread, especially among older colleagues. Moreover, the more aggressively you excavate, the more often you are confronted with situations that are classified as “caries profunda”, i.e., measures should then be taken to maintain pulp vitality in view of the thin residual dentin layer [8,15,16]. And this still happens millions of times a day in dental practices.

The next mystery is the theory behind IPC. It is assumed that at a residual dentin thicknesses of <300 microns, the barrier function of the dentin is reduced and it is advisable to apply agents that provoke dentin formation [14,17,18]. For decades, this was calcium hydroxide, which was said to stimulate new dentin formation due to its high pH [8]. But does this really work? In this context, it is interesting that glass ionomer cement is also mentioned in the current ESE guideline on the topic of IPC [8]. However, regarding their bioactive behavior, glass ionomer cement and calcium hydroxide have nothing in common [8,19]. So is it ultimately just a matter of covering deeper areas to prevent the penetration of resin tags when the dentin is bonded?

The purpose of this retrospective study was to evaluate the effect of IPC on the long-term clinical performance of restorations for 40 years, where the capping material was zinc–oxide–eugenol and the restorative material was amalgam in posterior teeth and resin composite in anterior teeth.

## 2. Materials and Methods

The study group was selected retrospectively from dental records of patients who underwent vital pulp therapy in a private dental practice and the research goes back as far as the year 1969. An investigation of 1412 dental records yielded 159 patients and 366 treated teeth. The 159 patients comprised 366 cases between 1969 and 1980. This means that an average of 2.3 (±1.58) teeth were treated per patient. The average year of birth was 1941. The average patient age at the beginning of the observation period was 26.5 (±9.24) years old. A total of 159 people were examined in the study. Of these, 82 were female (51.57%) and 77 were male (48.43%). The most frequently treated tooth in both the lower jaw (*n* = 61) and the upper jaw (*n* = 46) was the second permanent molar. Overall, the most frequently treated teeth were the first and second molars of the 4th quadrant with a frequency of 34 (9.29%) each.

Selected patients were treated by one experienced practitioner in the years between 1969 and 1980 and received indirect pulp capping. Inclusion criteria were vital permanent teeth with deep caries within the inner third of dentin, non-existent clinical signs of irreversible pulpitis, and no pulp exposure. Subsequent to the standardized ice-cold pulp-test, teeth were anaesthetized if required and cavities were prepared using a sterile high-speed bur with water coolant. Caries were excavated thoroughly with a round bur until the dentin showed increased resistance when streaked by a testing probe. Teeth with accidentally exposed pulp tissue were excluded. The remaining dentin layer was capped with zinc–oxide–eugenol cement according to manufacturer’s instructions. Posterior teeth were restored with amalgam fillings and anterior teeth received resin composite fillings after covering the zinc–oxide–eugenol capping with Harvard zinc phosphate cement.

The gathered parameters with possible influences on survival rates consisted of age, gender, tooth locations/positions, the dates of vital therapy, the number of filled surfaces, the types of primary restoration material, the successional treatments on each tooth, and the last date of surveillance. Data collection and statistical analysis were performed using Excel sheets (Microsoft Excel, Microsoft corporation, Redmond, WA, USA) and DataTab (DataTab, Seiersberg, Austria). The significant differences among groups were checked using cox regression analysis and the significance level was set at *p* = 0.05. Kaplan–Meier curves were utilised to illustrate survival rates. Treatment success was reached by the maintenance of vitality beyond 365 days. The loss of vitality within 365 days was determined as treatment failure. The outcome of treatment was assessed after different time periods (1 and 6 months; 1, 2, 5, 10, 20, and 40 years).

## 3. Results

### 3.1. Clinical Outcomes Within the First Year

**Figure 1:**
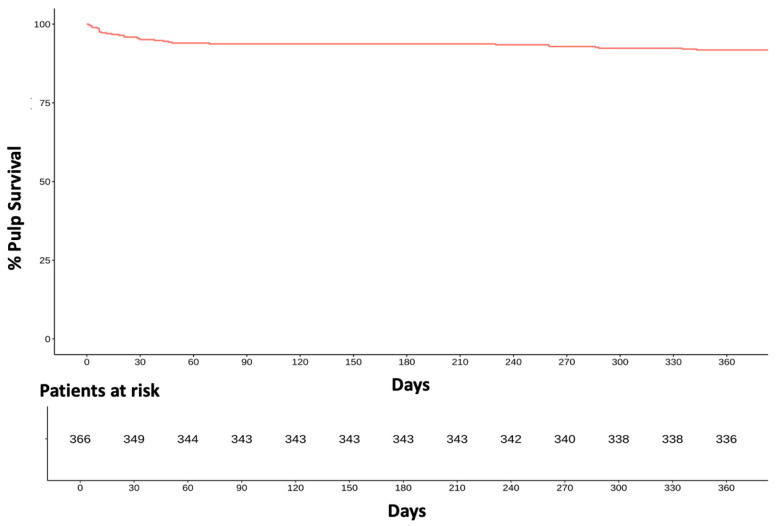
Development of pulp vitality with in the first year of observation.

A total of 336 of 366 indirectly capped pulps survived the first year after IPC. A detailed analysis of the survival rates within the first year after IPC showed an initial drop in the survival rates within the first 30 days. Here, the survival rate dropped to 95% after 30 days. A total of 343 of the 366 treated pulps (94%) survived the first three months. The survival rate saw a more pronounced drop during the first 10 days—from 100% to 97%. In the following 80 days, the survival rate dropped by a further 3%. In the further course, the survival rate dropped significantly more slowly (*p* < 0.05). After one year, the survival rate was 92%. (Figure 1). This means that the success rate of IPC in the present study was 92% because only failures until 12 months were previously defined as related to the IPC treatment.

### 3.2. Failures Within the First Year

**Figure 2:**
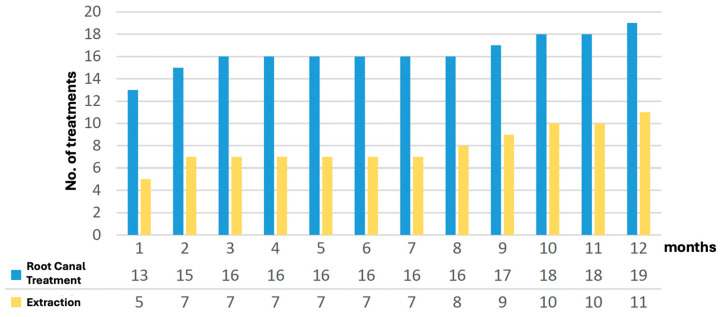
Interventions within the first year of observation.

The consequent treatments of failures within the first year, i.e., failures per definitionem, are displayed in Figure 2. Within the first month of observation, a total of 18 IPC treated teeth failed. Of these, 5 teeth were extracted and 13 teeth of the total 366 treated teeth received root canal treatment within the first month. Thus, 94.1% of the 366 treated teeth survived the first month. After three months, a total of 23 teeth (6.3%) had failed. During this period, 2 more teeth (out of the original 5) were removed and 4 more teeth (out of the original 12) received root canal treatment. Over the following four months, the failure rate remained static. During this period, no further teeth failed. From the 9th to the 12th month of observation, the failure rate increased to a total of 30 teeth (8.2%). The number of extractions and root canal treatments continued to rise steadily.

### 3.3. Overall Clinical Outcome

**Figure 3:**
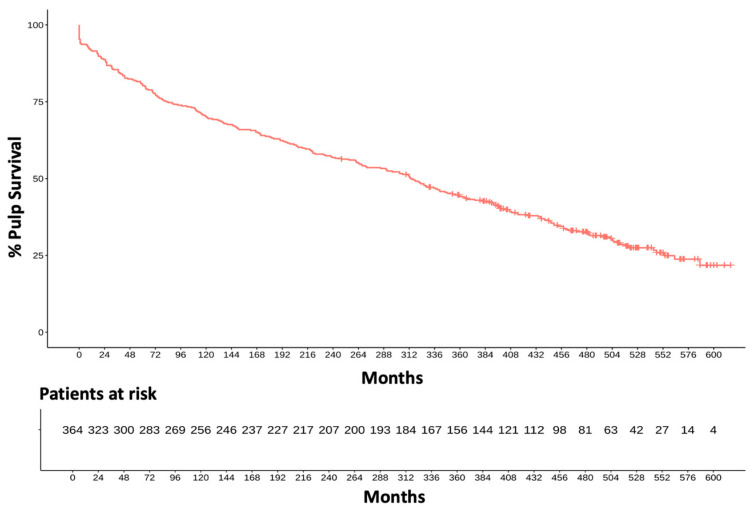
Development of pulp vitality over 40 years.

During the remaining 39 years of investigation, a linear occurrence of pulp vitality loss has been observed (Figure 2). The portions of vital pulps were tested to be 88% after two years, 80% after five years, 70% after 10 years, 57% after 20 years, and 32% after 40 years of clinical investigation resulting in an annual failure rate of 1.7% (Figure 3).

#### 3.3.1. The Overall Clinical Outcome Related to Cavity Size

**Figure 4:**
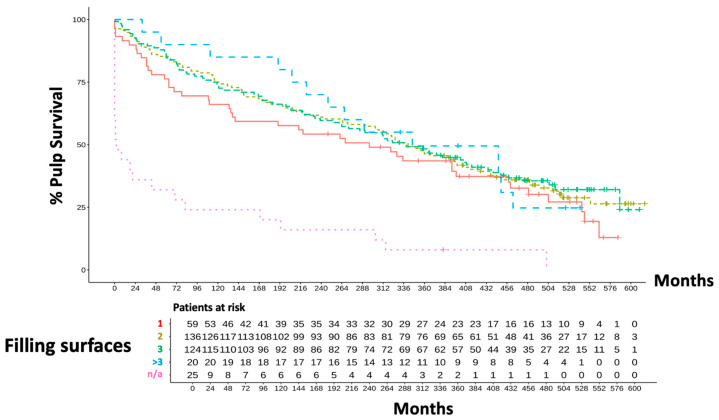
Overall clinical outcome related to cavity size.

The probability of a tooth with an IPC treatment and a subsequent two-surface filling surviving for 40 years was 36% (Figure 4). The survival probability of these teeth was thus significantly higher than that of teeth without information on the size of the filling (*p* < 0.05). In 25 of the 336 cases, no information was provided regarding the size of the filling or cavity. The cavities of these teeth were treated in their entirety with zinc–eugenol cement in order to test survival. These teeth showed a highly significant amount more failures (>50% after 2 years; *p* < 0.001). This confirmed a statistically significant difference in the survival curves based on cavity size. The cohort comparison of the filling sizes primarily showed that the 336 teeth examined most frequently received a 2—(*n* = 136) or 3—(*n* = 124) surface filling. Smaller, 1-surface fillings were placed about half as often over IPC (*n* = 59). Over the whole observation period of 40 years, cavity size had no negative influence (*p* = 0.063).

#### 3.3.2. The Overall Clinical Outcome Related to Tooth Position

**Figure 5:**
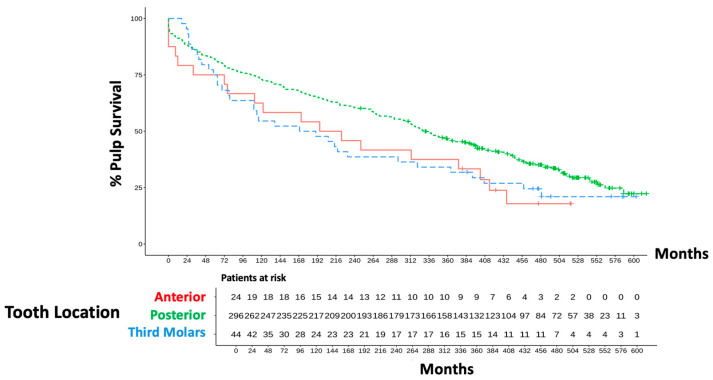
Overall clinical outcome related to tooth position.

Wisdom teeth initially appeared to have a higher survival rate (93% after 2 years). This value was relativized when considering the survival rates after 5 years to 66% (Figure 5). Over the course of time, the survival rates of wisdom teeth were inferior to those of posterior or anterior teeth. In the long-term analysis (40 years), the curve for the wisdom teeth fell between the survival rates of the front and side teeth. Posterior teeth have been the largest cohort. Here, the number of surviving teeth fell from 296 to 262 within the first 2 years (*p* = 0.774). Thus, no statistically significant differences could be derived with regard to the compared survival rates.

## 4. Discussion

The present retrospective practice-based study examined the long-term success of indirect capping prior to direct restorative therapy. Given the time period covered by this study, it is logical that the materials that were used are now considered outdated [2]. Nevertheless, the study serves the purpose of evaluating the long-term survival rates of pulp with reduced hard tissues, and this has not been done often in the past [20,21,22,23]. Of course, the 40-year study period is an advantage of the study, but the main disadvantage is its cross-sectional nature. Today, any kind of restorative therapy is characterized by a fundamentally different way of carrying out caries excavation. When the presented treatments have been carried out, of course the chosen excavation strategy was quite aggressive, i.e., extending in to the hypermineralized dentin layer providing maximum hardness [16]. Decades later, it has been clearly shown that selective excavation is preferable over the old-fashioned, so-called “complete” and usually aggressive excavation method [11,13,14]. However, also due to the age structure in dentist communities, seemingly complete excavation is still often practiced. Furthermore, the more a complete excavation is conducted, the more often “caries profunda” is present and consequently pulp vitality has to be preserved using IPC [21,24,25].

There are still different views and paradigms when it comes to the phenomenon of deep caries. However, there is always the risk that the vital pulp has already pathologically changed due to the effect of bacterial toxins from the carious biomass to such an extent that no indirect capping can stop or reverse this process towards irreversible pulpitis [17]. In such cases, it is generally irrelevant whether or not the decimated dentin near the pulp is covered. In theory, these cases should be excluded before the study takes place, but due to the rather stone age pulp diagnostics using cold, this is not possible. Today, it is considered relatively certain that the best way to ensure the vitality of the pulp is to seal the pulp–dentin complex as tightly as possible [3,4,8,26]. It is also certain that the best quality of the dentin sealing is achieved by an effective adhesive seal of the cavity floor in the dentin [1,5,14]. However, this raises the problem that in very deep cavities—we are talking about 300 μm and less residual dentin thickness—not only the hybrid layer creates adhesion, but resin tags also always arise independently of the bonding protocol [1]. However, the latter penetrate up to 300 μm into dentin, which means that these resin tags would then be located more or less at the entrance to the pulp in direct contact with the odontoblast layer, which is definitely not beneficial for a healthy pulp [27]. Regardless of the filling protocol, this leads to the necessity of covering these deep dentin areas [19]. Would it therefore be sufficient to cover the deep part of the cavity with a non-adhesive cement just to prevent the tags? This theory is at least discussed in the recent ESE guideline on this topic [8]. Although the present study will not be able to answer this question, it would definitely be interesting to explore whether modern techniques such as hydraulic cements or uncompromised adhesive dealing would perform better over such a long period of time. It should at least be mentioned that e.g., in posterior teeth there is no clinical difference between normal depth and deeper caries, for both ways a conventional lining is mandatory. Going the adhesive way on the other hand, although the primary goal is an optimal dentin seal, in the case of IPC a considerable amount of sealable dentin is covered with a non-adhesive material. This means that IPC using any kinds of cements fits far better into conventional methods compared to adhesive measures. From that clinical perspective, it would be desirable to develop ultra-mild, bioactive adhesives in order to solve both issues—IPC and maximum sealing capacity in one clinical step [2,10].

The second very widespread paradigm is to cover these deep cavity areas with a bioactive material to actively stimulate tertiary dentin formation, not least to reduce the influence on the pulp through dentin apposition [19,22,25,28,29]. For decades, calcium hydroxide was such a material [30]. In retrospect, the range of applications of calcium hydroxide can almost be considered a fairytale. However, it is not only the effectiveness of calcium hydroxide that is doubted today, but also the clinical handling of the soft pastes and, last but not least, the resorption rate [8]. Therefore, hydraulic cements are considered the material of choice today, but here too, it must be honestly noted that their effect on pulp exposure has been definitively proven, while the findings for indirect capping vary widely and in some cases light-curing calcium salicylate materials are also considered to be positive [20,24]. These facts lead to the assumption that in the end it really doesn’t matter what we use to cover this area, since “the seal is the deal” remains the primary success factor [3]. And this is precisely where the current study comes full circle. Regardless of whether deep dentin is covered with old-fashioned or “modern” materials, long-term clinical observations remain the key to gaining insights. And that is why the present study is of general interest to the field of restorative dentistry.

This retrospective, practice-based investigation demonstrates that even old-fashioned IPC using conventional zinc–eugenol cement as base material works out nicely over 40 years. It was a characteristic finding that apparently the first weeks after treatment determine whether the IPC treatment has been successful or not. Of course, several of these cases may have suffered the fact that irreversible pulp damage was already present without having been exactly diagnosable prior to the treatment. This is clearly visible when early losses of pulp vitality occurred. Another interesting finding is that the teeth having been labelled “n/a”, i.e., where expectative measures were taken into account, failed extremely often. Although the size of the carious lesion and consequently the size of the cavity did not appear to influence the long-term results, this was different at least during the vulnerable first phase of the observation period. Another aspect being of clinical interest is that the 40-years observation period of course was dominated by amalgam as restorative material for posterior teeth. Independent of the presence of IPC, conventional cement linings are always applied beneath amalgam, which has not changed up to now. However, in contrast to our study, in anterior teeth resin composite restorations are applied under a strict “total bonding” protocol without any cement lining. Therefore, it is not surprising that there is at least a tendency that anterior IPC restorations with presumably missing adhesive dentin seal performed inferiorly after all. Or to cut a long story short: the primary retention source for posterior amalgam restorations have been undercuts provided by preparation; however, the primary retention scenario in anterior teeth was solely bonding to enamel margins, because the dentin was covered with cements completely due to the lack of effective dentin adhesives back then.

So what is finally the decisive factor for the successful treatment of deep caries? From today’s knowledge, it seems to be advisable to selectively remove caries in order not even get close to the question whether IPC is necessary at all [11,12,13,16,31,32]. For the case of old-school methods, seemingly complete caries excavation (which fakes sterile conditions and actually never happens [13]) also conventional cements like in the present study may be successful. For the case of fully adhesive restorations such as direct resin composites, it is finally also important that IPC materials are compatible with consequent bonding procedures instead of corroborating them via substantial contamination of the bonded surface [26]. Altogether, except for the crucial first year of observation, the other 30 years of clinical performance exhibited a quite linear behavior of pulp vitality loss.

## 5. Conclusions

IPC using zinc–oxide–eugenol cement showed excellent long-term success after 40 years of clinical service. The predominant number of failures after IPC occurred during the first three months of clinical service. In this vulnerable period, larger defects as well as expectative measures led to significantly more frequent losses of pulp vitality.

## Data Availability

Data are available on reasonable request.
